# Validation of the French Version of the World Health Organization Quality of Life HIV Instrument

**DOI:** 10.1371/journal.pone.0073180

**Published:** 2013-09-03

**Authors:** Gregory Reychler, Gilles Caty, Anne Vincent, Simon Billo, Jean-Cyr Yombi

**Affiliations:** 1 Institut de Recherche Expérimentale et Clinique (IREC), Pôle de Pneumologie, ENT & Dermatologie, Université Catholique de Louvain, Brussels, Belgium; 2 Service de Pneumologie, Cliniques universitaires Saint-Luc, Brussels, Belgium; 3 Département de Médecine Physique et Réadaptation, Cliniques universitaires Saint-Luc, Brussels, Belgium; 4 Centre de prise en charge VIH, Cliniques universitaires Saint-Luc, Brussels, Belgium; University of Ottawa, Canada

## Abstract

**Purpose:**

Quality of life is a key element in the follow-up of people living with HIV/AIDS. The main purpose of this study was to validate the French version of the WHOQOL-HIV instrument by comparing this instrument to a generic questionnaire. The second objective was to test the reproducibility of this questionnaire.

**Method:**

The WHOQOL-HIV and SF-36 questionnaires were filled out by 50 patients on two separate occasions with a time interval of 2 weeks. The internal consistency, validity and reliability of the WHOQOL-HIV were evaluated.

**Results:**

The internal consistency was acceptable for the different domains, with Cronbach’s alpha ranging from 0.937 to 0.944. The facet-domain correlations were all statistically significant (p<0.001). There was a correlation between the domains from the WHOQOL-HIV and SF-36 questionnaires, with coefficients ranging from 0.349 to 0.763 (p<0.05 for all), except for the *Spirituality* domain. The test-retest reliability was suitable for all domains and facets, with statistically significant intra-class coefficients between 0.615 and 0.931.

**Conclusion:**

This study demonstrated that the French translation of the WHOQOL-HIV instrument is a valid and reproducible tool for the evaluation of the quality of life for HIV-infected patients.

## Introduction

Currently, UNAIDS estimates that more than 35 million people are infected with human immunodeficiency virus (HIV) around the world, and more than 10% of this population is living in French-speaking countries [1].

It has been well established that in chronic diseases, an emerging element is the evaluation of the quality of life (QoL) [2]. Moreover, the QoL is a primary outcome of many studies [3]. Even though the overall HIV epidemic trends are declining, as long as there is no curative treatment for HIV/AIDS, people will continue to suffer from the disease, and measurement of the quality of life will remain a key factor in the outcome of these patients. Moreover, the life expectancy of HIV-infected patients has increased due to the efficacy of highly active antiretroviral treatment (HAART) [4]. With this enhancement of life expectancy, people living with HIV/AIDS are facing more co-morbidities, such as osteoporosis, cardiovascular disease and renal impairment.

The examination of the relationship between HIV infection and the QoL has been increasingly studied over the last 10 years. For example, the QoL was investigated in populations from different countries [5]–[10] and in older adults with HIV infection [11]. The QoL in HIV infection was also compared to the QoL in other chronic diseases [12]. It has been reported that the QoL is related to CD4^+^ cell counts [13], [14], the onset of symptoms [12], [14], depression and stress [15], [16] and antiretroviral treatment [17].

A reliable instrument to measure QoL is necessary, and many generic questionnaires exist. In chronic diseases, there is a particular need for disease-specific questionnaires evaluating the QoL. Initially, the World Health Organization defined the concept of the quality of life and developed a questionnaire to specifically assess the quality of life for HIV-infected patients (World Health Organization Quality of Life-HIV instrument (WHOQOL-HIV)) [18]. The development of this questionnaire was completed by a multi-centric pilot test in 8 different countries [18]–[20].

The evaluation of the validity and reliability of this questionnaire is extremely important. Moreover, to be effective in a specific country, such an instrument must be translated and validated in the local language. Although this instrument was translated into other languages [21]–[25], it has not been translated in French.

The aim of this study was to validate the French version of the WHOQOL-HIV instrument by comparing this instrument to a generic questionnaire. Furthermore, the test-retest reliability of the French version was tested at 2 weeks.

## Materials and Methods

### Ethics Statement

The study was approved by the regional Ethics Committee in Cliniques universitaires Saint-Luc and Université catholique de Louvain in Brussels (B403201213334). All of the patients provided written informed consent.

### Subjects

HIV-infected patients regularly attending the outpatient infectious disease clinic of Cliniques universitaires Saint-Luc were recruited on a voluntary basis and without financial compensation for this study. Consecutive patients fulfilling the inclusion criteria were selected by the physician after approval of the patients to enroll in the study until 50 patients were included in the second part of the study.

The following inclusion criteria were used: 18 years or older, infected with HIV for at least 6 months [26] and a native French speaker (patients born in a francophone family speaking French at home and living in the francophone part of Belgium). The patients that were unstable (defined by any modification of health outcomes) for the duration of the study were excluded from the second phase of the study.

### Protocol

The study included two phases. During the visit, the patients received both questionnaires (WHOQOL-HIV and MOS 36-item short form (SF-36)). They were asked to fill out these questionnaires while attending the consultation (Phase I). During this time, they received a second package including the questionnaires with a stamped and addressed reply envelope. They were asked to fill out these questionnaires 15 days later and then send them back so that the responses obtained during the initial visit could be compared with the later responses (Phase II). If more than 20% of the items were not filled out, the patient was excluded from the study. Trained researchers were present to provide assistance in completing the questionnaires if necessary.

### Procedure

The process was based on Beaton’s guidelines [27]. The initial WHOQOL-HIV instrument was translated from English into international French using two bilingual translators with a medical background whose primary language was French. Both French translations were performed, and any resulting issues were resolved. One independent translator translated this new version back into English, and the translation was compared to the original version. This translator was a native English speaker.

### Questionnaires

#### WHOQOL-HIV

WHOQOL-HIV is a self-reported questionnaire. In the original version, socio-demographic information regarding sex and age are obtained. The HIV-related information includes the mode of transmission, HIV status and year of diagnosis. General health status is evaluated by asking the subjects to rate his or her health on the Likert scale, which ranges from very poor (1) to very good (5). WHOQOL-HIV includes 120 items and 37 important questions. The structure of the WHOQOL-HIV questionnaire includes a profile with scores across six domains (physical, psychological, level of dependence, social relationships, environment and spirituality) and 29 facets, with 5 of these facets relating to HIV/AIDS (symptoms of person living with HIV/AIDS (PLWHA), social inclusion, forgiveness and blame, concerns about the future, death and dying). Non-specific questions concerning the subject’s overall QoL and health status are also included. All of the items are rated on a five-point scale (*not at all* to *extremely* for the intensity and capacity domains; *never* to *always* for frequency; *very dissatisfied* to *very satisfied* or *very good* to *very poor* for evaluation). For negatively framed items, the scores are reversed so that the higher the score, the better the QoL. A score is calculated from the facet and domain. Each item of a facet and facet of a domain contribute equally to the facet and domain scores, respectively.

#### MOS 36-item short form (SF-36)

The SF-36 is an auto-evaluation short-form health instrument [28], [29] and was previously validated in French [30]. This form includes 36 items and covers eight different scales: physical functioning (PF), role limitations due to physical health problems (RP), bodily pain (BP), general health perceptions (GH), vitality (VT), social functioning (SF), role limitations related to emotional problems (RE), and mental health (MH). A linear transformation on a 0 to 100 scale (the lower the score, the worse the status) was performed. The physical component summary (PCS) and mental component summary (MCS) were also calculated by aggregating the 8 previous scales.

### Statistical Analysis

The sample size needed (n = 52) to compare WHOQOL-HIV to SF36 (Phase I) was determined by the method for studies involving linear regression [31] with a power of 80.

The data were computed using SPSS 20.0 (IBM software) for Windows. A descriptive analysis was performed for the socio-demographic and HIV-related information. The internal consistency was assessed using Cronbach’s alpha. The correlation between the domains and overall quality of life was calculated for WHOQOL-HIV. The validity was measured using Pearson correlations to determine the strength of the relationship between the scores for the hypothesised interrelated domains from the two instruments and between each item and its domain for the WHOQOL-HIV. The hypothesised interrelation was based on two expert opinions. The experts were involved in quality of life evaluation (CG) and in the follow-up of patients living with HIV/AIDS (YJC) respectively. Their final decisions were based on their own opinion and consensual. The test-retest reliability was evaluated using an intra-class coefficient (ICC) for agreement, and agreement was estimated using the Bland Altman method. The correlations between the variables were measured using ICCs [32]–[34]. The ICCs determine the extent of the relative discrepancies between the evaluations and give the proportion of variance attributable to differences between the groups. The effect size was calculated for each domain and item by taking the difference between the mean baseline and follow-up scores on the measurement and dividing by the standard deviation of the baseline scores. All of the tests were two-tailed with a statistical significance level fixed at a p value of 0.05.

## Results

Among the 64 enrolled subjects in the first phase, 50 were included in phase II of the study. The patients who were not included either did not complete or did not return the questionnaires or were hospitalised during this period. The socio-demographic data of the patients are summarised in Table 1. The age range of the patients was 20 to 66 years old (mean age = 41.2±10.9 yrs). The mean CD4^+^ cell count was 605.4/mm^3^. Eighty-four percent of the patients had undetectable viral loads, and 90% of the patients were undergoing HAART. Sixteen percent of the patients were classified as CDC stage C. Neither questionnaire contained more than 20% of missing responses.

**Table 1:** Socio-demographic relatives and related-HIV informations of the sample of patients.

	Group test-retest
**Gender**	
Male	32 (64.0)
Female	18 (36.0)
**Education**	
None	0 (0.0)
Primary school	0 (0.0)
Secondary school	22 (44.0)
Second cycle	20 (40.0)
Third cycle	8 (16.0)
**Marital status**	
Single	21 (42.0)
Married/Co-habiting	17 (34.0)
Separeted/Divorced	8 (16.0)
Widowed	4 (8.0)
**Health status**	
Poor	2 (4.0)
Neither good nor poor	13 (26.0)
Good	27 (54.0)
Very good	8 (16.0)
**HIV status**	
Asymptomatic	39 (78.0)
Symptomatic	3 (6.0)
AIDS	8 (16.0)
**Treatment history**	
HAART	45 (90.0)
Untreated	5 (10.0)
**Route of infection**	
Sexual intercourse with man	36 (72.0)
Sexual intercourse with woman	3 (6.0)
Drug use	1 (2.0)
Blood contamination	5 (10.0)
Other	5 (6.0)

Results are expressed by number of subjects (percentage).

The scores for the different facets and domains for the WHOQOL-HIV instrument, correlations and internal consistency values are presented in Table 2. WHOQOL-HIV demonstrated acceptable internal consistency for the different domains, with Cronbach’s alpha values ranging from 0.937 (Psychological) to 0.944 (Spirituality) (Table 2). The Cronbach’s alpha values were acceptable for all of the facets (higher than 0.70) (Table 2). The facet-domain correlations ranging between 0.538 and 0.905 were all statistically significant (p<0.001) (Table 2).

**Table 2:** Descriptive scores for WHOQOL-HIV.

	Mean	SD	Correlation	Cronbach
**Domain 1 _ Physical**	14.60	3.57	–	0.942
Pain and discomfort	3.72	0.91	0.720	0.941
Energy and fatigue	3.48	0.92	0.843	0.940
Sleep and rest	4.00	1.00	0.632	0.941
Symptoms of PLWHAs	3.61	1.05	0.765	0.940
**Domain 2 _ Psychological**	14.89	2.54	–	0.937
Positive feelings	3.68	0.75	0.812	0.941
Cognitions	3.79	0.68	0.840	0.941
Self-esteem	3.63	0.69	0.842	0.941
Body image and appearance	3.70	1.01	0.722	0.941
Negative feelings	3.81	0.83	0.815	0.941
**Domain 3 _ Level of independence**	15.78	3.30	–	0.940
Mobility	4.23	0.88	0.751	0.941
Activities of daily living	4.10	0.81	0.844	0.940
Dependence on medication or treatment	3.40	1.36	0.705	0.941
Work capacity	4.04	1.04	0.905	0.940
**Domain 4 _ Social relationships**	14.74	2.54	–	0.940
Personal relationships	3.94	0.59	0.796	0.941
Social support	3.54	0.80	0.723	0.942
Sexual activity	3.40	1.03	0.731	0.941
Social inclusion	3.87	0.92	0.803	0.941
**Domain 5 _ Environment**	15.38	2.20	–	0.938
Physical safety and security	3.78	0.69	0.728	0.941
Home environment	3.89	0.84	0.760	0.941
Financial resources	3.37	1.08	0.735	0.941
Health and social care	4.19	0.57	0.542	0.942
New information or skills	3.92	0.71	0.633	0.942
Recreation and leisure	3.64	0.77	0.823	0.941
Physical environments	3.98	0.60	0.702	0.942
Transport	4.00	0.90	0.747	0.941
**Domain 6 _ Spirituality**	14.11	3.37	–	0.944
Spirituality, Religion, Personal beliefs	3.38	1.25	0.538	0.944
Forgiveness	3.81	1.07	0.713	0.941
Fear of the future	3.53	1.13	0.835	0.941
Death and dying	3.41	1.19	0.833	0.941
**Overall QoL & general health perception**	15.56	2.82	–	0.938

The analysis of the score distribution in our patient population revealed an absence of ceiling and floor effects for all of the domains of WHOQOL-HIV with less than 15% of the patients with a highest or lowest possible score, respectively. The ceiling effects were nearly achieved (14%) for the item *Overall QoL and health status*. No floor effects were observed, with less than 10% of the patients having the lowest scores for all domains.

The results of the validation process revealed statistically significant correlations between the overall quality of life and different domains of WHOQOL-HIV, with all of the coefficients ranging from 0.418 (Spirituality) to 0.735 (Environment). The correlations between all of the domains for this instrument were significant, with the exception of the correlation between *Spirituality* and *Environment* (p = 0.119) (Table 3). Moreover, significant correlations for all of the hypothesised interrelated domains between SF-36 and WHOQOL-HIV were observed (Table 3).

**Table 3:** Coefficients of correlation between domains of WHOQOL-HIV and SF-36.

	WHOQOL-HIV							SF-36							
	D1	D2	D3	D4	D5	D6	D suppl	BP	GH	VT	SF	MH	RE	RP	PF
D1	1							0.544		0.763					
D2	0.662	1										0.703			
D3	0.538	0.650	1										0.667	0.667	0.699
D4	0.394	0.660	0.595	1							0.511				
D5	0.565	0.695	0.723	0.660	1						0.595				
D6	0.521	0.537	0.355	0.320	0.224	1						0.540			
D suppl	0.613	0.714	0.683	0.677	0.735	0.418	1		0.583						

D1 = physical domain, D2 = psychological domain, D3 = independence domain, D4 = social domain, D5 = environment domain, D6 = Spirituality, D suppl = Overall QoL, BP = bodily pain, GH = general health perceptions, VT = vitality, SF = social functioning, MH = mental health, RE = role emotional, RP = role physical, PF = physical functioning.

**Figure 1:**
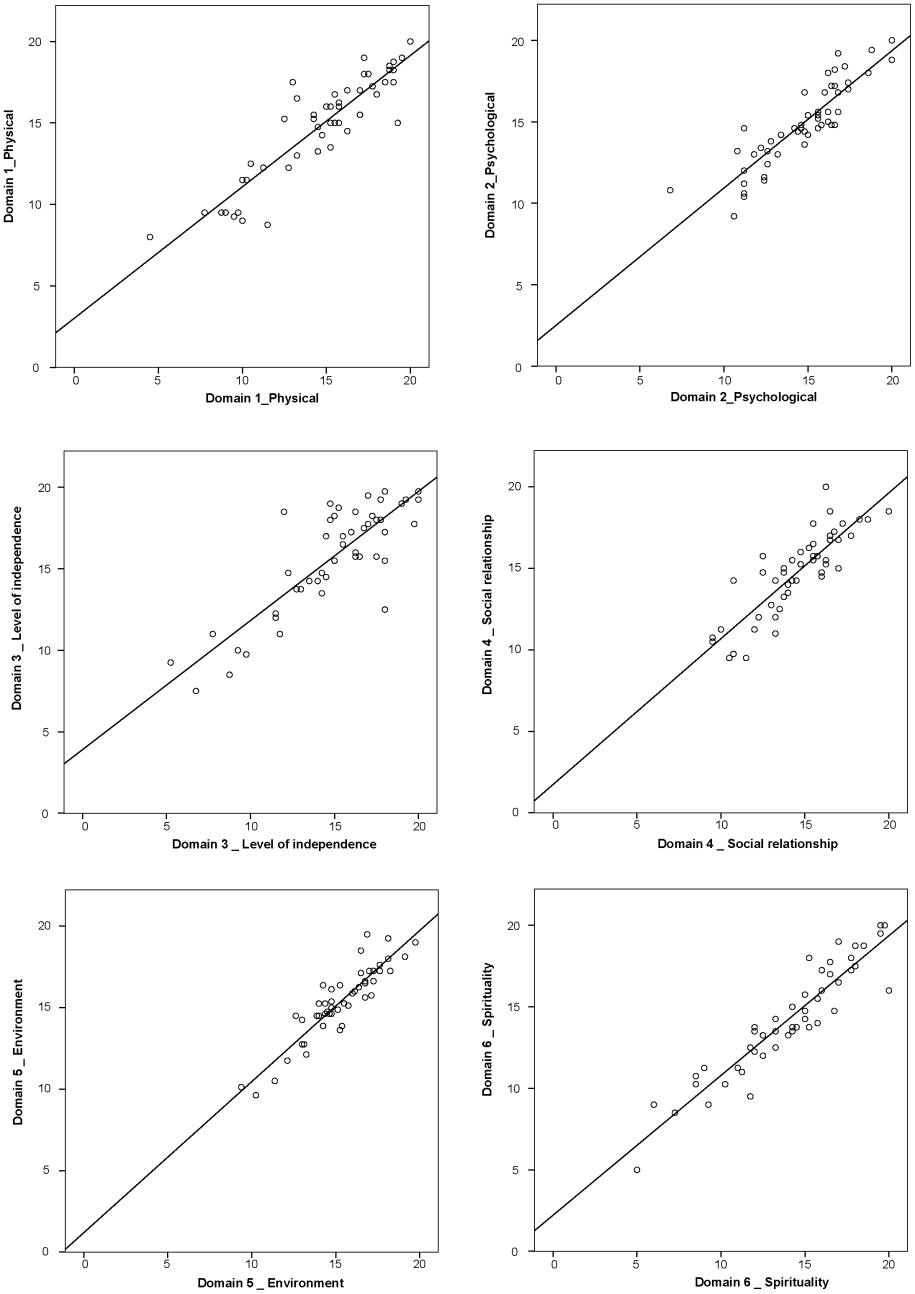
Relationship between scores of domains for test-retest WHOQOL-HIV.

The test-retest reliability contained statistically significant intra-class coefficients for all of the domains and facets (Table 4) (Figure 1). The Bland Altman method revealed a difference between the measurement days for the domains (−0.015 to 0.795) and the facets (−0.110 to 0.260). All of the differences were included between the limits of agreement. Finally, the effect size was small for all of the items and domains, varying from 0 to 0.36.

**Table 4:** Results for the WHOQOL-HIV at day 1 and day 15 and intra-class coefficient (ICC) for the different items and domains of the WHOQOL-HIV for the test-retest validation.

	*WHOQOL-HIV day 1*		*WHOQOL-HIV day 15*		*Mean bias*		*Limits of agreement*		*ICC*
	Mean	SD	Mean	SD	Mean	SD	Low	High	
**Domain 1 _ Physical**	14.60	3.57	14.61	3.58	−0.015	2.274	−4.472	4.442	0.798
Pain and discomfort	3.72	0.91	3.76	0.99	−0.045	0.550	−1.123	1.033	0.832
Energy and fatigue	3.48	0.92	3.40	0.98	0.075	0.382	−0.674	0.824	0.919
Sleep and rest	4. 00	1.00	3.88	1.03	0.120	0.637	−1.129	1.369	0.802
Symptoms of PLWHAs	3.61	1.05	3.57	1.18	0.035	0.747	−1.430	1.500	0.776
**Domain 2 _ Psychological**	14.89	2.54	14.70	2.67	0.192	1.249	−2.257	2.641	0.885
Positive feelings	3.68	0.75	3.61	0.69	0.075	0.563	−1.028	1.178	0.696
Cognitions	3.79	0.68	3.72	0.64	0.070	0.385	−0.684	0.824	0.828
Self-esteem	3.63	0.69	3.57	0.67	0.060	0.470	−0.861	0.981	0.761
Body image and appearance	3.70	1.01	3.70	1.06	0	0.578	−1.133	1.133	0.845
Negative feelings	3.81	0.83	3.79	0.96	0.025	0.426	−0.811	0.861	0.887
**Domain 3 _ Level of independence**	15.78	3.30	14.98	3.51	0.795	1.925	−2.978	4.568	0.840
Mobility	4.23	0.88	4.04	0.97	0.185	0.701	−1.190	1.560	0.715
Activities of daily living	4.10	0.81	3.99	0.87	0.110	0.485	−0.840	1.060	0.835
Dependence on medication or treatment	3.40	1.36	3.14	1.48	0.260	1.184	−2.062	2.582	0.652
Work capacity	4.04	1.04	3.82	0.99	0.220	0.686	−1.125	1.565	0.770
**Domain 4 _ Social relationships**	14.74	2.54	14.54	2.41	0.205	1.361	−2.462	2.872	0.849
Personal relationships	3.94	0.59	3.72	0.65	0.215	0.395	−0.558	0.988	0.796
Social support	3.54	0.80	3.55	0.78	−0.015	0.686	−1.359	1.329	0.624
Sexual activity	3.40	1.03	3.37	0.98	0.030	0.658	−1.259	1.319	0.786
Social inclusion	3.87	0.92	3.90	0.86	−0.025	0.635	−1.270	1.220	0.744
**Domain 5 _ Environment**	15.38	2.20	15.31	2.15	0.073	0.951	−1.791	1.936	0.905
Physical safety and security	3.78	0.69	3.81	0.76	−0.030	0.573	−1.153	1.093	0.690
Home environment	3.89	0.84	3.82	0.72	0.060	0.453	−0.829	0.949	0.833
Financial resources	3.37	1.08	3.34	0.99	0.020	0.494	−0.949	0.989	0.886
Health and social care	4.19	0.57	4.20	0.53	−0.020	0.448	−0.899	0.859	0.665
New information or skills	3.92	0.71	3.95	0.63	−0.025	0.587	−1.176	1.126	0.615
Recreation and leisure	3.64	0.77	3.57	0.74	0.060	0.526	−0.971	1.091	0.756
Physical environments	3.98	0.60	3.80	0.63	0.180	0.388	−0.581	0.941	0.802
Transport	4.00	0.90	4.11	0.79	−0.110	0.547	−1.182	0.962	0.793
**Domain 6 _ Spirituality**	14.11	3.37	13.86	3.67	0.240	1.308	−2.324	2.804	0.931
Spirituality, Religion, Personal beliefs	3.38	1.25	3.30	1.29	0.070	0.567	−1.041	1.181	0.900
Forgiveness	3.81	1.07	3.60	1.13	0.200	0.732	−1.235	1.635	0.777
Fear of the future	3.53	1.13	3.54	1.16	−0.005	0.538	−1.060	1.050	0.889
Death and dying	3.41	1.19	3.42	1.26	−0.015	0.644	−1.277	1.247	0.862
**Overall QoL & general health perception**	15.56	2.82	15.10	2.84	0.460	1.876	−3.217	4.137	0.781

All ICC are statistically significant (p<0.001).

## Discussion

Based on the distribution of scores for the WHOQOL-HIV questionnaire, we report good internal consistency and validity. Moreover, the test-retest validity was verified.

The internal consistency was excellent for all of the items and domains, with Cronbach’s alpha scores ranging from 0.937 to 0.944. Internal consistency is classically considered acceptable for Cronbach’s alpha between 0.70 and 0.95 [35]. Moreover, our results are similar [21], [36], [37] or even better [25] than the validations of this questionnaire in other languages.

Pearson’s correlation coefficients between individual items and their respective domains were also calculated to assess the structural validity of the questionnaire. Values greater than 0.30 are associated with an acceptable result [38]. The structural validity of the questionnaire is very good, with all of the coefficients higher than 0.50. Only the *health and social care* (0.542) and *spirituality, religion, personal beliefs* (0.538) items showed a slightly lower reliability than for the other items.

Ceiling and floor effects occur when 15% or more of the patients respond with a highest or lowest score, respectively [39]. Similar to the Italian [24], Portuguese [21] and Brazilian [25] versions, we did not observe these effects for any domain in our patients. Therefore, the validity is acceptable with discriminative extreme values.

The validity of the WHOQOL-HIV questionnaire is favourable. Indeed, the WHOQOL-HIV scores are related to the SF-36 scores for the interrelated domains. Even though a significant correlation was found between *spirituality* and *general health perception*, the hypothesised interrelationship between these two domains should be discussed. The concepts included in the *spirituality* domain are not fully integrated in the SF-36 questionnaire. However, it has been previously demonstrated that the *spirituality* item is not as important for HIV patients compared to other components of the quality of life [21].

The test-retest reliability was evaluated by an intra-class coefficient for agreement, which is the most suitable reliability parameter [35] and more adequate than the intra-class coefficient for consistency [32]. Both instruments were administered again at two weeks. Two weeks is a time period that is long enough to prevent the patients from remembering the previous test but short enough to avoid changes in health status. As a value of 0.70 represents the minimum standard for reliability [40], our results demonstrated a good test-retest reliability for all of the domains. Moreover, using the Bland and Altman method, our results reveal a bias lower than 0.800 for all of the domains. The Bland and Altman method [41] is an adequate method to observe absolute measurement errors between two repetitive tests [35].

The effect size is defined as small (ES <0.2), small to moderate (ES between 0.2 and 0.5), moderate to large (ES between 0.51 and 0.79), and large (ES >0.79) [42]. The effect size is a simple way to quantify the size of the difference between two measurements. In our study, nearly all of the effect sizes were lower than 0.2. Only three effect sizes were between 0.2 and 0.4. These results highlight good agreement between the measurements.

The limitation of this study is that the sample better reflects the quality of life for patients followed in a health care centre rather than other patients. However, the characteristics of the sample are comparable to the Belgian HIV population according to the last report from the Belgian Scientific Institute of Public Health (2010) [43]. The ratio between men and women who took part in the study is 1.77 compared to 1.7 in the Belgium population of HIV-infected patients, and the CD4^+^ T cell counts are greater than the mean Belgian levels. Moreover, the mean age and percentage of treated patients are only slightly higher (37.6 vs 41.2 yrs and 80 vs 90%, respectively) than the global population in our centre. Finally, responsiveness remains to be tested. However, all of the psychometric properties of a scale cannot be established in a single study [44].

In conclusion, the French version of WHOQOL-HIV is valid for the evaluation of the quality of life for HIV patients. This questionnaire correlates with a well-validated questionnaire (SF-36). Moreover, the French version of WHOQOL-HIV is a reliable tool with good reproducibility within a two-week period. Specificity and sensitivity have been previously demonstrated [36].

The French version of the questionnaire can be downloaded here: http://www.saintluc.be/services/medicaux/vih/WHOQOL-HIV-questionnaire.pdf.
